# Supplementary material for: Axl, Immune Checkpoint Molecules and HIF Inhibitors from the Culture Broth of Lepista luscina
Source: Molecules. 2022 Dec 15;27(24):8925. doi: 10.3390/molecules27248925 (PMC9781456; doi:10.3390/molecules27248925)
Supplement: Supplementary file 1 [file molecules-27-08925-s001.zip › molecules-2056002-supplementary.pdf]

molecules

*Supplementary Data*

# Axl, Immune Checkpoint and HIF Inhibitors from Culture Broth of *Lepista luscina*

**Mihaya Kotajima [1,†], Jae-Hoon Choi [1,2,3,4,†], Mitsuru Kondo [1,2], Corina N. D'Alessandro-Gabazza [5], Masaaki Toda [5], Taro Yasuma [5], Esteban C. Gabazza [5],Yukihiro Miwa [6], Chiho Shoda [6], Deokho Lee [6], Ayaka Nakai [6], Toshihide Ku-rihara [6], Jing Wu [3,4], Hirofumi Hirai [1,2,3,4] and Hirokazu Kawagishi [3,4,*]**

[1] Graduate School of Science and Technology, Shizuoka University, 836 Ohya, Suruga-ku, Shizuoka 422-8529, Japan
[2] Research Institute of Green Science and Technology, Shizuoka University, 836 Ohya, Suruga-ku, Shizuoka 422-8529, Japan
[3] Faculty of Agriculture, Shizuoka University, 836 Ohya, Suruga-ku, Shizuoka 422-8529, Japan
[4] Research Institute for Mushroom Science, Shizuoka University, 836 Ohya, Suruga-ku, Shizuoka 422-8529, Japan
[5] Department of Immunology, Mie University Graduate School of Medicine, Edobashi 2-174, Mie 524-8507, Japan
[6] Department of Ophthalmology, Keio University School of Medicine, 35 Shina-nomachi, Shinjuku-ku, Tokyo 160-8582, Japan

* Correspondence: kawagishi.hirokazu@shizuoka.ac.jp
† These authors contributed equally to this work.

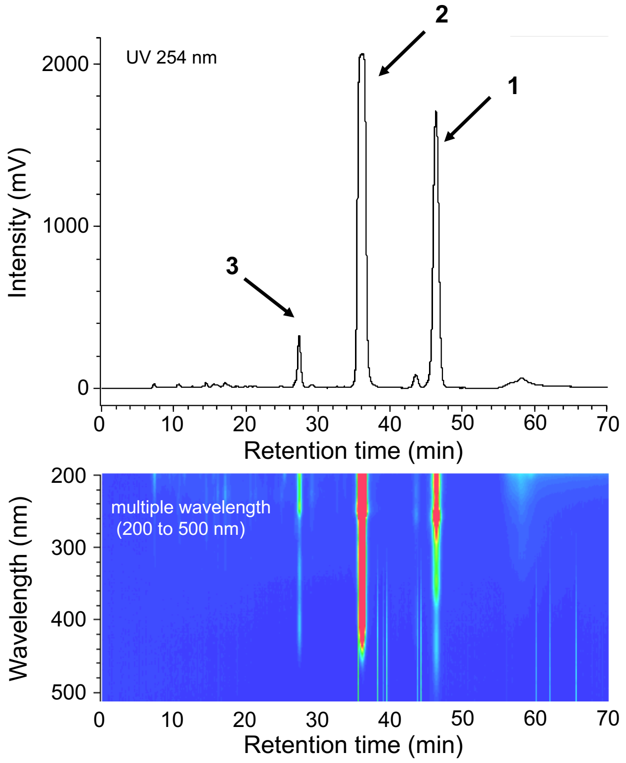


Figure S1. HPLC profile of metabolites from hexane soluble part of liquid culture of *L. luscina*.

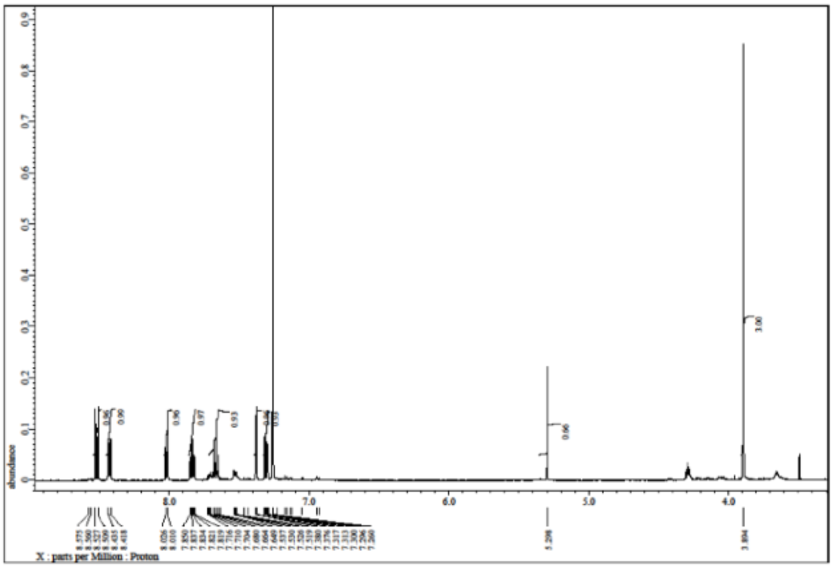


Figure S2. $^{1}H$ NMR spectrum of **1** ($CDCl_3$).

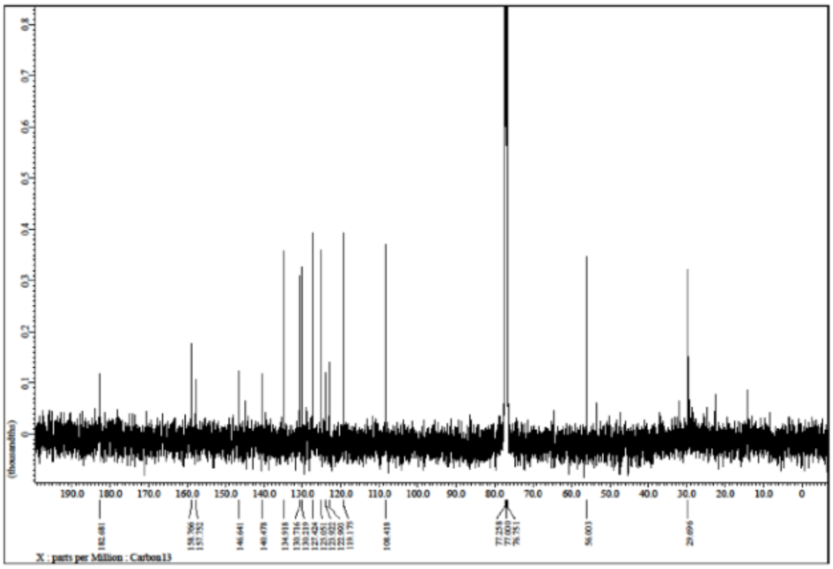

Figure S3. $^{13}C$ NMR spectrum of **1** ($CDCl_3$).

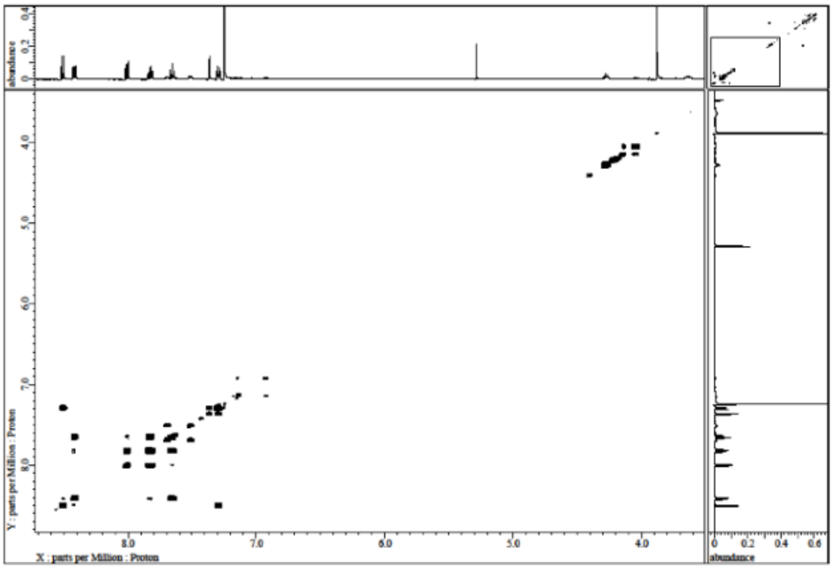

Figure S4. COSY spectrum of **1** ($CDCl_3$).

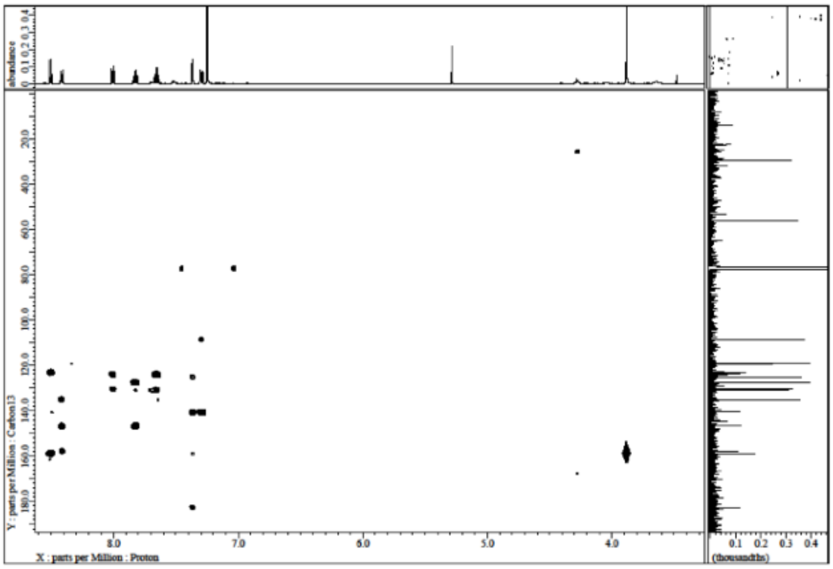


Figure S5. HMBC spectrum of **1** ($CDCl_3$).

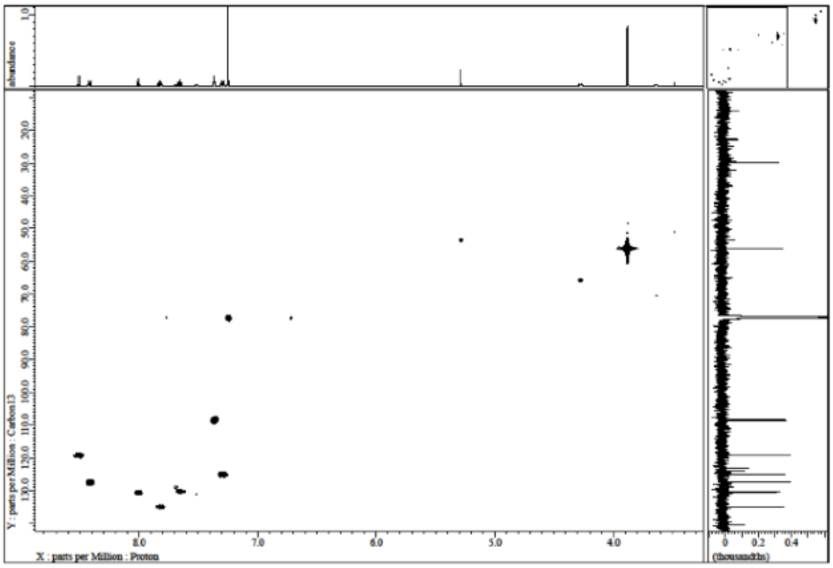


Figure S6. HMQC spectrum of **1** ($CDCl_3$).

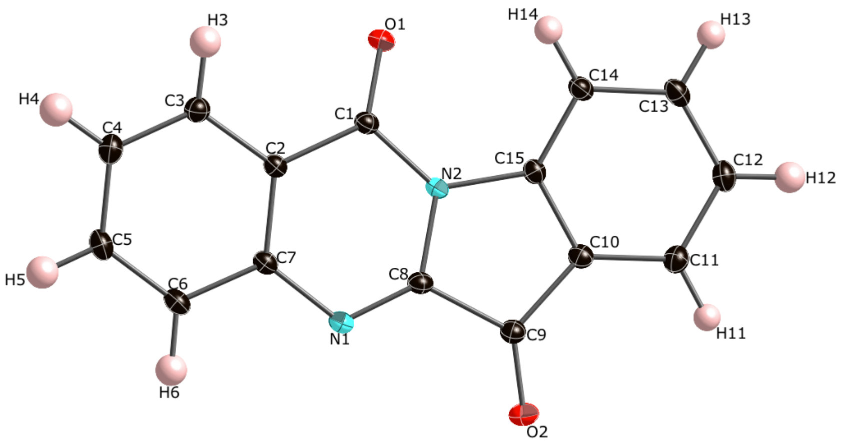


Figure S7. ORTEP drawings of **2** with ellipsoids at the 50% probability level.

**Table S1.** Crystalloraphic table of **2**.

| Empirical formula | $C_{15}H_8N_2O_2$ |
|---|---|
| Formula weight | 248.23 |
| Temperature/K | 173.15 |
| Crystal system | monoclinic |
| Space group | $P2_1/n$ |
| $a$/Å | 7.3022 (2) |
| $b$/Å | 7.5655 (2) |
| $c$/Å | 19.4313 (5) |
| $\alpha$/° | 90 |
| $\beta$/° | 91.160 (2) |
| $\gamma$/° | 90 |
| Volume/Å$^3$ | 1073.26 (5) |
| $Z$ | 4 |
| $\rho_{calc}$ g/cm$^3$ | 1.536 |
| $\mu$/mm$^{-1}$ | 0.105 |
| $F$(000) | 512.0 |
| Crystal size/mm$^3$ | 0.34 × 0.15 × 0.1 |
| Radiation | Mo $K\alpha$ ($\lambda$ = 0.71073) |
| Reflections collected | 21807 |
| Independent reflections | 2908 [$R_{int}$ = 0.0283, $R_{sigma}$ = 0.0199] |
| Data/restraints/parameters | 2908/0/204 |
| Goodness-of-fit on $F^2$ | 1.063 |
| Final R indexes [$I > \sigma (I)$] | $R_1$ = 0.0464, $wR_2$ = 0.1154 |
| Final R indexes [all data] | $R_1$ = 0.0551, $wR_2$ = 0.1196 |
| Largest diff. peak/hole / e Å$^{-3}$ | 0.45/-0.21 |

A suitable crystal was selected and on a Rigaku VariMax Saturn CCD (1.2 kW Mo rotating anode) at 173 K. The structure was solved with the SHELXT and refined with the SHELXL refinement package using Least Squares minimization[48,49]. The structure analyzed was essentially the same with the paper previously reported[50].

48. Sheldrick, G. M. *SHELXT* - Integrated space-group and crystal-structure determination. *Acta Crystallographica Section A* **2015**, *71*, 3-8, doi:10.1107/S2053273314026370.

49. Sheldrick, G. M. Crystal structure refinement with *SHELXL*. *Acta Crystallographica Section C* **2015**, *71*, 3-8, doi:10.1107/S2053229614024218.
50. Fedeli, W.; Mazza, F. J. Crystal structure of tryptanthrin (indolo[2,1-*b*]quinazoline-6,12-dione). *Journal of the Chemical Society, Perkin Transactions 2* **1974**, *13*, 1621-1623, doi:10.1039/P29740001621.
